# Chronic intracranial EEG recordings and interictal spike rate reveal multiscale temporal modulations in seizure states

**DOI:** 10.1093/braincomms/fcad205

**Published:** 2023-07-19

**Authors:** Gabrielle M Schroeder, Philippa J Karoly, Matias Maturana, Mariella Panagiotopoulou, Peter N Taylor, Mark J Cook, Yujiang Wang

**Affiliations:** CNNP Lab (www.cnnp-lab.com), Interdisciplinary Computing and Complex BioSystems Group, School of Computing, Newcastle University, Newcastle upon Tyne NE4 5TG, UK; Graeme Clark Institute and St Vincent’s Hospital, University of Melbourne, Parkville, Victoria 3010, Australia; Department of Biomedical Engineering, University of Melbourne, Parkville, Victoria 3010, Australia; Graeme Clark Institute and St Vincent’s Hospital, University of Melbourne, Parkville, Victoria 3010, Australia; Department of Biomedical Engineering, University of Melbourne, Parkville, Victoria 3010, Australia; Research Department, Seer Medical Pty Ltd., Melbourne, Victoria 3000, Australia; CNNP Lab (www.cnnp-lab.com), Interdisciplinary Computing and Complex BioSystems Group, School of Computing, Newcastle University, Newcastle upon Tyne NE4 5TG, UK; CNNP Lab (www.cnnp-lab.com), Interdisciplinary Computing and Complex BioSystems Group, School of Computing, Newcastle University, Newcastle upon Tyne NE4 5TG, UK; Faculty of Medical Sciences, Newcastle University, Newcastle upon Tyne NE1 7RU, UK; UCL Queen Square Institute of Neurology, Queen Square, London WC1N 3BG, UK; Graeme Clark Institute and St Vincent’s Hospital, University of Melbourne, Parkville, Victoria 3010, Australia; CNNP Lab (www.cnnp-lab.com), Interdisciplinary Computing and Complex BioSystems Group, School of Computing, Newcastle University, Newcastle upon Tyne NE4 5TG, UK; Faculty of Medical Sciences, Newcastle University, Newcastle upon Tyne NE1 7RU, UK; UCL Queen Square Institute of Neurology, Queen Square, London WC1N 3BG, UK

**Keywords:** epilepsy, seizure variability, circadian, multidien, intracranial EEG

## Abstract

Many biological processes are modulated by rhythms on circadian and multidien timescales. In focal epilepsy, various seizure features, such as spread and duration, can change from one seizure to the next within the same patient. However, the specific timescales of this variability, as well as the specific seizure characteristics that change over time, are unclear. Here, in a cross-sectional observational study, we analysed within-patient seizure variability in 10 patients with chronic intracranial EEG recordings (185–767 days of recording time, 57–452 analysed seizures/patient). We characterized the seizure evolutions as sequences of a finite number of patient-specific functional seizure network states. We then compared seizure network state occurrence and duration to (1) time since implantation and (2) patient-specific circadian and multidien cycles in interictal spike rate. In most patients, the occurrence or duration of at least one seizure network state was associated with the time since implantation. Some patients had one or more seizure network states that were associated with phases of circadian and/or multidien spike rate cycles. A given seizure network state’s occurrence and duration were usually not associated with the same timescale. Our results suggest that different time-varying factors modulate within-patient seizure evolutions over multiple timescales, with separate processes modulating a seizure network state’s occurrence and duration. These findings imply that the development of time-adaptive treatments in epilepsy must account for several separate properties of epileptic seizures and similar principles likely apply to other neurological conditions.

## Teaser

Schroeder *et al*. report that seizure evolutions vary across chronic intracranial EEG recordings, with many changes associated with circadian and multidien fluctuations in interictal spike rate. These findings suggest that different time-varying factors modulate seizure features over multiple timescales.

## Graphical Abstract

**Graphical abstract:**
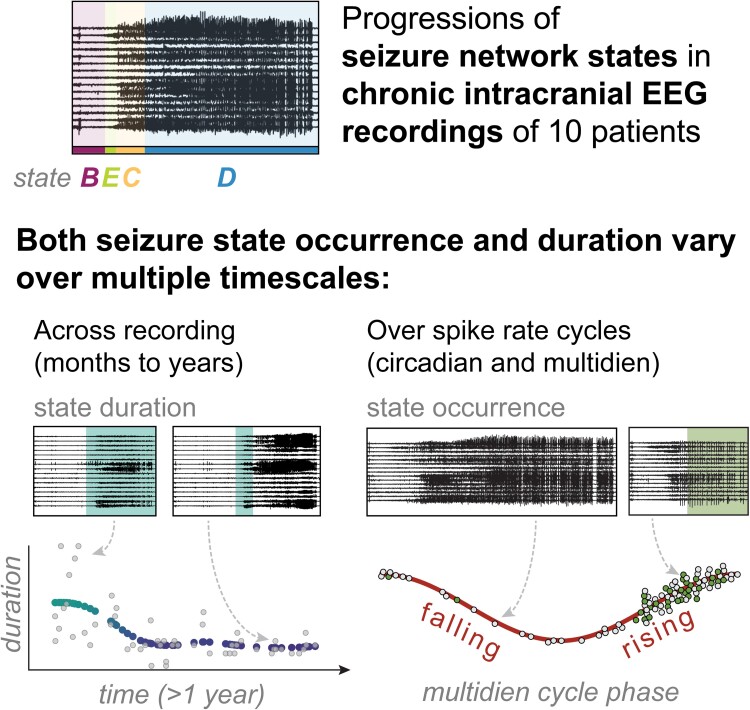

See O. Baud and R. Rao (https://doi.org/10.1093/braincomms/fcad230) for a scientific commentary on this article.

## Introduction

Focal epilepsy is characterized by recurrent, unprovoked seizures. Importantly, these seizures are not homogeneous events, even in the same patient. Within individual patients, seizure features such as clinical symptoms,^1^ onset locations and patterns,^2-6^ duration,^7,8^ and network evolutions^9^ can change over time and potentially influence treatment responses.^7,10,11^ As such, a better understanding of the patterns and sources of within-patient seizure variability is needed.

One open question is whether and how such seizure features change over short (e.g. 24 h) and long (e.g. weekly, monthly and yearly) periods of time. There is some evidence that seizures are modulated over such timescales. Certain clinical seizure types and symptoms, such as focal to bilateral tonic-clonic seizures, can preferentially occur during specific parts of sleep/wake or day/night cycles.^12-16^ Electrographic seizure onset patterns can shift across the days of epilepsy monitoring unit^3^ and months of chronic intracranial EEG (iEEG)^17^ recordings, suggesting that seizure features can also change over slower timescales. To quantify within-patient variability in seizure dynamics, we recently compared seizure functional network evolutions,^9^ which capture the time-varying relationships (e.g. correlation or coherence) between the activity of different brain regions from seizure start to end. This description of seizure activity builds on the concept of epilepsy as a network disorder,^18,19^ and seizure network features have been linked to seizure symptoms,^20-22^ seizure termination,^23,24^ and the seizure onset zone.^20,25,26^ Our preliminary analysis of epilepsy monitoring unit patients found that seizure network evolutions do not change randomly over time.^9^ Instead, in most patients, the changes in seizure network evolutions could be explained by a combination of circadian and/or slower time-varying factors. However, these temporal associations and the specific timescales and seizure network changes need to be characterized in longer recordings with larger numbers of seizures.

In recent years, chronic iEEG recordings over months to years have provided unprecedented insights into epileptic brain dynamics over multiple timescales.^27-30^ First, these recordings have revealed fluctuations in interictal dynamics, including in the rates and spatial patterns of bursts,^17^ spikes,^31^ high-frequency activity,^31^ and other signal features.^32^ This variability is especially high in the first months after electrode implantation, possibly due to the brain’s response to acute trauma.^17,31,32^ However, more persistent variability in such features has also been observed,^17,31^ suggesting that other mechanisms also drive the observed interictal shifts. In addition, multiple studies have found prevalent patient-specific circadian, multidien (multiday) and/or circannual cycles in interictal features and seizure occurrence.^31,33-37^ Since the exact periods of these cycles often vary over time, they are best tracked using fluctuations in continuous biomarkers such as interictal spike rate.^34,35,37^ An intriguing possibility is that seizure characteristics could also change over such cycles. However, the relationship between seizure features and spike rate cycles has not been explored.

We addressed these questions by analysing changes in seizure networks in chronic iEEG recordings from the NeuroVista (NV) dataset.^28^ We follow a popular approach of describing seizures as a sequence of a small number of patient-specific functional network states,^20,25^ each of which described a recurring relationship between the recorded brain areas. Compared to our previous descriptions of seizure network evolutions,^8,9^ this states-based approach allowed us to easily identify and compare which network patterns occurred in each seizure. In each patient, we then analysed changes in seizure network states over multiple timescales. We first identified gradual changes in seizure network states across the course of each recording. We then determined if seizure network states also fluctuated over patient-specific circadian and multidien cycles that were revealed by interictal spike rate. To account for possible independent variability in seizure evolutions and seizure duration,^8^ we separately examined variability in seizure network state occurrence and seizure network state duration. We show that in most patients, both of these features were associated with multiple timescales, providing new insight into the patterns and possible mechanisms of within-patient seizure variability.

## Methods

In the following, we summarize our methods, while Supplementary Methods provide detailed descriptions of the analyses.

### Patients and seizure data

We analysed seizure data from 10 NV patients that underwent chronic iEEG recordings.^28^ The seizure recordings are part of a dataset of 12 patients that was previously made available by Ref. ^38^. From the original cohort, patients NV 2 and NV 4 were excluded from our analysis due to low numbers of recorded seizures (32 and 22 seizures, respectively). All other patients had at least 57 analysable seizures. The patients and collection of their chronic iEEG data are described in detail in Ref. ^28^, and patient details are provided in Supplementary Table 1. Anonymized data was analysed under the approval of the Newcastle University Ethics Committee (reference number 6887/2018). The original data acquisition is detailed in Ref. ^28^, and ‘the human research ethics committees of the participating institutes approved the study and subsequent amendments. All patients gave written informed consent before participation’.^28^

Seizures were annotated by clinical staff after identification using patient diaries, audio recordings and a seizure detection algorithm.^7^ Seizures with clinical manifestations and corresponding iEEG changes (‘type 1’ seizures) and seizures with iEEG changes comparable to type 1 seizures, but without confirmed clinical manifestations (‘type 2’ seizures) were included in the analysis.^7,38^ We excluded seizures with noisy segments (identified visually) and duration less than 10 s.

### Computing progressions of seizure network states

After re-referencing the iEEG data to a common average reference, we obtained the sliding window (10 s window, 9 s overlap) functional connectivity (band-averaged coherence) in six frequency bands: delta 1–4 Hz, theta 4–8 Hz, alpha 8–13 Hz, beta 13–30 Hz, gamma 30–80 Hz and high gamma 80–150 Hz. To characterize each patient’s seizure evolutions, we soft-clustered the windows in time using non-negative matrix factorization (NMF),^39^ allowing us to assign each time window a ‘seizure network state’ (SNS). The number of SNSs in each patient was determined using stability NMF.^40^ This approach, which we have previously applied to seizure iEEG data,^9^ takes advantage of the nondeterministic nature of NMF to find which number of SNSs always produces the same set of SNSs. We then described seizure network evolution as a sequence, or progression, of SNSs. Note that NMF does not produce orthogonal SNSs, allowing some overlap in connectivity patterns between SNSs (see Supplementary Fig. 1).

### Defining SNS features

In each patient, we investigated two types of variability in SNSs: SNS occurrence and SNS duration.

SNS occurrence was a binary seizure feature defined as whether a given SNS occurred in the seizure. Most SNSs did not occur in all of a patient’s seizures; thus, these SNS had variable occurrence. We excluded SNSs that occurred in all seizures from the SNS occurrence analysis.

SNS duration was a continuous measure that quantified the number of time windows a seizure spent in a given SNS. For each SNS, we analysed SNS duration in all seizures containing that SNS; in other words, zero durations were excluded from the analysis to avoid effects driven by SNS occurrence. We analysed this feature for all SNSs.

### Defining temporal features

We compared the SNS features of each patient’s seizures to two types of temporal features: time since implantation and spike rate cycle phase.

A seizure’s time since implantation was defined as the number of days after the recording’s start that the seizure occurred. This measure captured changes in SNSs over the course of the entire iEEG recording.

To analyse SNSs over shorter timescales, we first extracted fluctuations in the interictal epileptiform spike rate. The continuously recorded spike rate of each patient was detected and validated in a previous study.^41^ We then extracted the prominent circadian and multidien (multiday) fluctuations in spike rate using empirical mode decomposition (EMD)^42-44^ (See Supplementary Methods for details). EMD is a data-driven technique that decomposes a time series into a series of oscillatory components that can have variable amplitude and frequency over time, thus accommodating non-stationarity in the spike rate cycles. We limited our analysis to the most prominent components that had short periods relative to the length of the patient’s recording. We refer to each component as a ‘spike rate cycle’ throughout the rest of the paper. For each patient, we then extracted the phases at which each seizure occurred, thus assigning each seizure a phase for each spike rate cycle. Seizures were excluded from this analysis if they occurred during a time period with insufficient spike rate cycle data (see Supplementary Methods).

### Statistical analysis

For each patient, we compared their SNS features (SNS occurrence and duration) to each temporal feature (time since implantation and the patient’s spike rate cycle phases).

For each SNS with the variable occurrence, a Wilcoxon rank sum test was used to compare the time since the implantation of seizures with and without the SNS. To quantify the temporal separation of seizures with and without the SNS, the area under the curve (AUC) of the receiver operating characteristic curve for distinguishing SNS occurrence using seizure times was also computed. Note that AUCs are mathematically equivalent to the Wilcoxon rank sum test statistic and therefore have the same statistical significance.

To compare SNS duration to seizure time since implantation, we computed the Spearman correlation between non-zero SNS durations and seizure times. We used the corresponding test statistic to determine the statistical significance of the association.

To compare SNS occurrence to spike rate cycles, for each spike rate cycle we determined the phase preference of seizures with the SNS by computing the mean resultant vector from the seizure phases:


$$Re^{-i\psi }=\frac{1}{S}\overset{S}{\underset{s=1}{\sum }}\;e^{-i\phi_{s}}$$

Here, *S* is the number of seizures containing the SNS, *s* is a seizure with the SNS, $\phi_{s}$ is the spike rate cycle phase of seizure *s*, *R* is the modulus of the mean resultant vector, and ψ is the angle of the mean resultant vector. As in previous work,^34,35^ we refer to *R* as the phase locking value (PLV) of seizures containing the SNS. The PLV varies from 0 to 1 and is higher when the cycle’s phases are similar across all seizures with the SNS. To control for any seizure phase preference, we used permutation tests to determine the significance of the observed PLV for each SNS and spike rate cycle. For a SNS that occurred *S* times, we randomly selected *S* seizures from all analysed seizures and recomputed the PLV. Repeating this process for 10 000 different permutations yielded a null distribution of PLVs for the scenario that the SNS had no additional phase preference within that spike rate cycle. The *P*-value of the SNS’s phase preference was defined as the percentage of times a permuted PLV was greater than or equal to the observed PLV.

Finally, we compared SNS duration to each spike rate cycle. For each cycle and SNS, we computed the rank linear-circular correlation *D* between the SNS’s duration and the cycle phases of the corresponding seizures.^45^*D* varies from 0 to 1, with higher values indicating a stronger association between the SNS’s duration and the spike rate cycle phases. We determined the significance of these associations by randomly shuffling the seizures’ SNS duration 10 000 times and computing a null distribution of correlations. The *P*-value of the observed correlation was the percentage of times a permuted correlation was greater than or equal to the observed correlation.

Benjamini–Hochberg false discovery rate (FDR) correction for multiple comparisons, with α=0.05, was performed for all tests across all patients that compared seizure features (SNS occurrence and SNS duration) to temporal features (seizure time in the recording and spike rate cycles), and only significant results are reported. Uncorrected *P*-values are reported in the text for reference.

## Results

### Seizure network evolutions vary from seizure to seizure within individual patients

**Figure 1:**
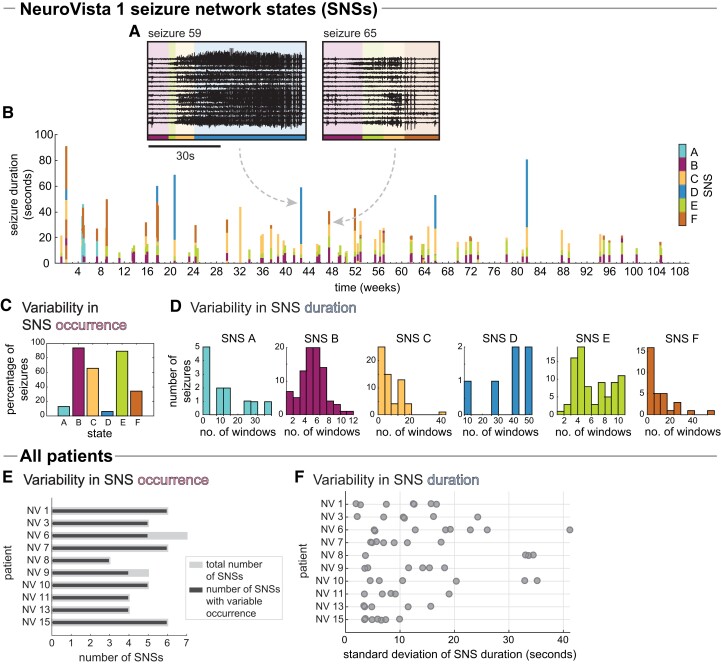
**Variability in seizure network state (SNS) progressions.** (**A–D**) Example patient, NeuroVista (NV) 1: (**A**) iEEG of two example seizures, with their corresponding SNSs overlaid. (**B**) All seizures: each seizure is represented by a vertical bar; its horizontal location indicates the time of seizure occurrence and its height indicates the seizure’s duration. The colours of the bar indicate the SNS. (**C**) Percentage of seizures that contained a given SNS in NV 1. (**D**) Histograms of SNS duration in NV 1. Seizures that do not contain a given SNS are excluded from the corresponding histogram. (**E, F**) All patients: (**E**) The number of SNSs in each patient (light grey bars), with the number of those SNSs that had variable occurrence (i.e. did not occur in all of the patient’s seizures) overlaid with dark grey bars. (**F**) Variability in SNS duration for each patient. Each dot corresponds to a single SNS.

In each patient, we first characterized changes in seizure network evolutions over the course of each patient’s continuous iEEG recording. Specifically, we described seizure network evolutions as a sequence, or progression, of seizure network states (SNSs). Figure 1A shows the SNS progressions of two example seizures in patient NV 1. Both seizures began with the same three SNSs (B, E and C), but had different final SNS (D in seizure 59 versus F in seizure 65). Visually, the amplitude and frequency of the seizure activity also differ once their SNS progressions diverge. Supplementary Fig. 1 provides the complete SNS visualizations of NV 1 to visualize the information captured by each SNS.

Figure 1B shows all of NV 1’s SNS progressions. It is already visually apparent that SNS progression differed from seizure to seizure. In particular, there was variability in both SNS occurrence (Fig. 1C) and SNS duration (Fig. 1D). For example, SNS D only occurred in 6.4% of seizures, and, in those seizures, its duration could range from 8 to 52 windows.

Across patients, we also observed variability in SNS progressions (Fig. 1E and F, and see Zenodo Data File 10.5281/zenodo.5910238 for data for all patients). Importantly, SNS are not comparable between patients in our study due to the patient-specific intracranial implantation schemes, which record from different brain areas in each patient. Since both SNS occurrence and duration varied across all patients, we investigated both features in the following.

### Seizure network states vary over the duration of chronic iEEG recordings

We first asked if within-patient SNS occurrence and duration varied over the timescale of each patient’s entire chronic iEEG recording. Specifically, we explored whether seizures that occurred early in the recording had different features from those seizures that occurred later.

**Figure 2:**
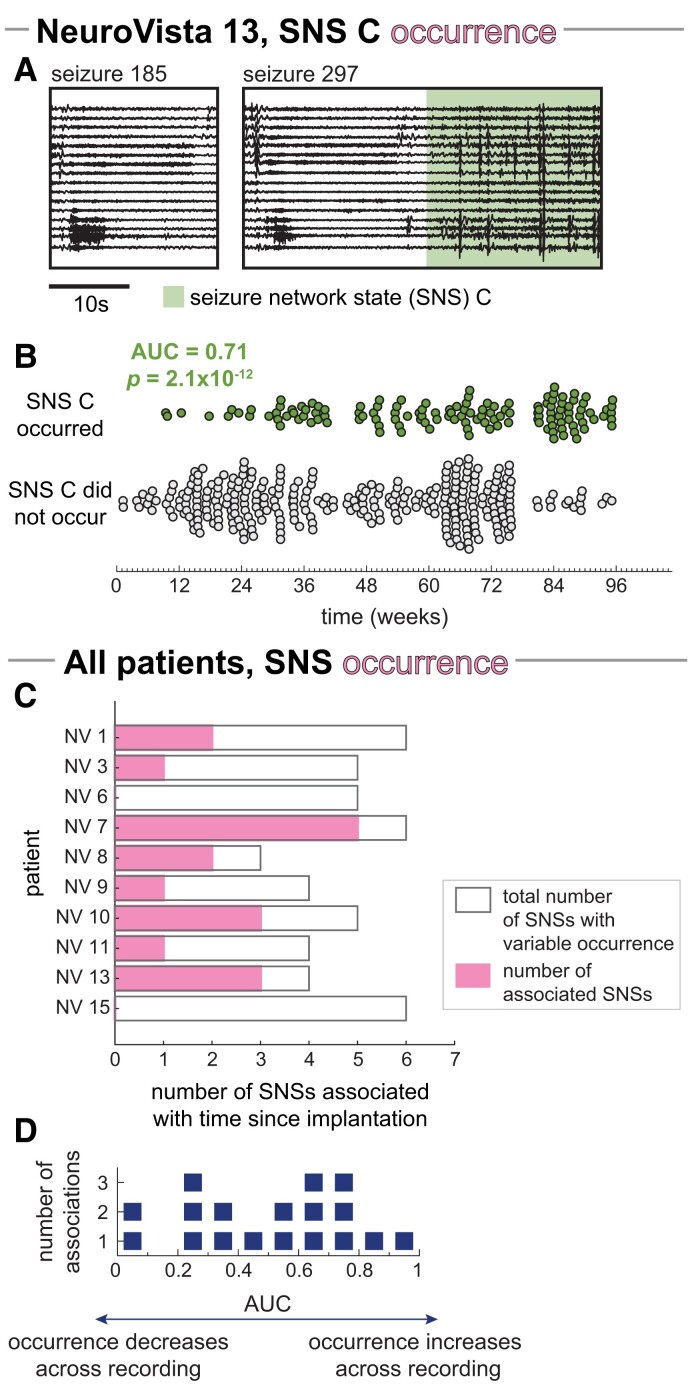
**Relationship between seizure network state (SNS) occurrence and time since implantation.** (**A, B**) Example patient, NeuroVista (NV) 13: (**A**) Example iEEG of seizures with and without SNS C (highlighted with green). Both seizures begin similarly, but only seizure 297 progresses further to SNS C. (**B**) Time since the implantation of seizures with (green circles) and without (grey circles) SNS C. Points are spread vertically to prevent overlap between seizures with similar times. Time since implantation separated seizures with and without SNS C with an AUC of 0.71. C-D) All patients: (**C**) Number of SNSs with significant associations with time since implantation in each patient (pink bars). Bar outlines provide a reference for the maximum possible number of associated SNSs (i.e. the number of SNSs with variable occurrence, equivalent to the dark grey bars in Fig. 1F). (**D**) Dot plot of the AUCs for each significant SNS in (**C**). Each marker corresponds to one SNS.

We first investigated relationships between the amount of time elapsed since the iEEG implantation and SNS occurrence. Figure 2A and B show the relationship of an example SNS with recording time. In this patient, NV 13, only some seizures contained SNS C (Fig. 2A) and these seizures tended to occur towards the end of the recording period. The temporal separability of seizures with and without SNS C can be characterized by the AUC, with an AUC below 0.5 indicating that the SNS preferentially occurred in earlier seizures and an AUC above 0.5 revealing that the SNS tended to occur in later seizures. Here, SNS C has an AUC of 0.71, which was significant after FDR correction for multiple comparisons (Wilcoxon rank-sum test, $p=2.1\times 10^{-12}$).

Across our cohort, eight out of the ten patients had at least one SNS where the SNS’s occurrence was significantly associated with the time since implantation (Fig. 2C). These temporal associations were not driven by transient SNSs that only occurred during the initial part of the recording (Supplementary Fig. 2).

**Figure 3:**
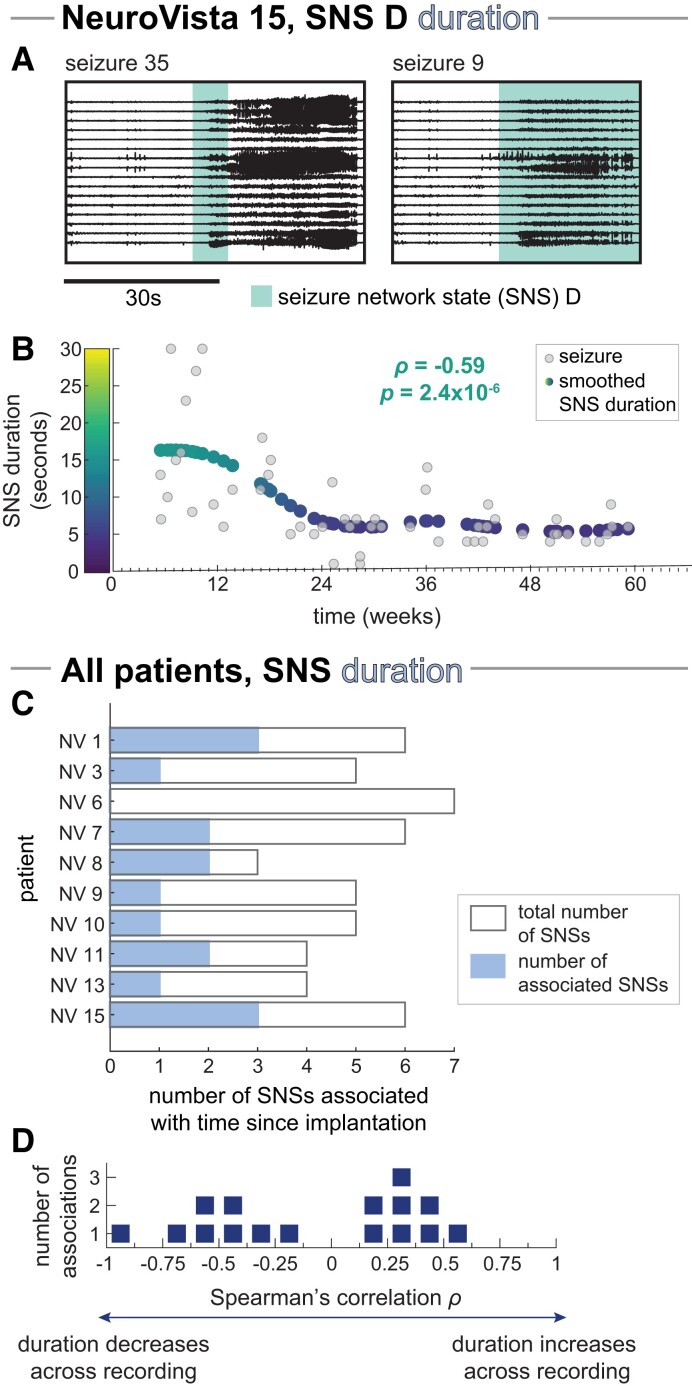
**Relationship between SNS duration and time since implantation.** (**A, B**) Example patient, NeuroVista (NV) 15: (**A**) Example iEEG of seizures with SNS D (highlighted with teal). Seizure 35 briefly visits SNS D before progressing to another SNS, while seizure nine lingers in SNS D before terminating. (**B**) SNS D duration versus the time since implantation, with a smoothed trend line (Gaussian window of 24 weeks) shown with the coloured points. Each light grey point corresponds to a seizure that contained SNS D. (**C, D**) All patients: (**C**) Number of SNSs with significant associations with recording time in each patient (blue bars). Bar outlines provide a reference for the maximum possible number of associated SNSs (i.e. the total number of SNSs in each patient, equivalent to the light grey bars in Fig. 1E). (**D**) Dot plot of the Spearman’s correlation between SNS duration and recording time for all significantly associated SNSs. Each marker corresponds to one SNS. For each SNS, we only analysed SNS duration in seizures containing the SNS, thus ensuring that our results were not driven by seizures without the SNS.

NV 15’s SNS D demonstrates how SNS duration can also vary over the length of the recording (Fig. 3A and B). Here, SNS D’s duration was significantly higher in earlier seizures, as demonstrated by a significant Spearman’s correlation ρ of −0.59 after FDR correction for multiple comparisons ($p=2.4\times 10^{-6}$).

Almost all patients had at least one SNS where the SNS’s duration was either significantly positively (eight SNSs) or negatively (eight SNSs) correlated with time since implantation (Fig. 3C and D). These findings demonstrate that, across a patient’s chronic iEEG recording, it is also possible for an SNS’s duration to increase or decrease.

### Seizure network states fluctuate over circadian and multidien cycles

Next, we hypothesized that SNSs, like seizure occurrence,^34-37^ may vary over circadian and multidien cycles. Importantly, these cycles can be non-stationary, with the cycle period varying over time; thus, they must be extracted using a continuous biomarker such as interictal spike rate.^34-37^

**Figure 4:**
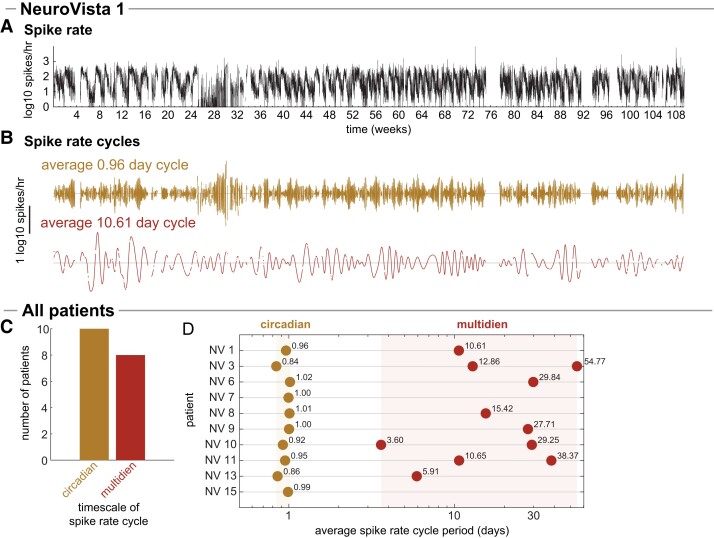
**Patient-specific cycles in interictal spike rate.** (**A, B**) Example patient NeuroVista (NV) 1: (**A**) The interictal spike rate (number of spikes per hour versus recording time) during NV 1’s recording. (**B**) The two prominent cycles in NV 1’s spike rate, were extracted using EMD. NV 1 had a circadian (average period of 0.96 days) and multidien (average period of 10.61 days) cycle. (**C, D**) All patients: (**C**) Number of patients that had at least one spike rate cycle at each timescale. (**D**) Spike rate cycles of each patient, coloured by their timescale.

As in previous work,^34-37,41^ we observed high levels of variability in interictal spike rate across each patient’s chronic iEEG recording (see Fig. 4A for interictal spike rate of an example patient, NV 1). We obtained each patient’s interictal spike rate from a previous study^41^ and used a data-driven approach, EMD,^42-44^ to extract prominent spike rate cycles over different timescales (see Zenodo Data File 10.5281/zenodo.5910238 for spike rate decompositions of all patients). EMD can extract cycles with variable periods, which is a common characteristic of multidien spike rate cycles.^34-37^ To approximate each spike rate cycle’s period, we report each cycle’s average period across the iEEG recording. Figure 4B shows the extracted spike rate cycles of NV 1. We observed such multiscale fluctuations in interictal spike rate in most patients (Fig. 4C and D, see Supplementary Fig. 3 for selection of spike rate timescales). All patients had prominent circadian cycles in spike rate (average period of 0.84–1.02 days), and eight of the ten patients also had at least one multidien cycle, with average periods ranging from 3.60 to 54.77 days. Together, these cycles characterized the prominent patient-specific, non-stationary changes in spike rate in each patient.

For each patient, we first asked whether a given SNS preferentially occurred during certain phases of each spike rate cycle. Similar to previous work,^33-35^ we defined phase preference as the PLV of a SNS for a spike rate cycle. A PLV of 0 would indicate that the SNS had no phase preference, while a PLV of 1 would indicate that the SNS only occurred at one specific phase of the cycle. Importantly, seizures themselves usually have phase preferences for circadian and multidien spike rate cycles.^34-37^ Therefore, to control for seizure timing phase preferences, we used permutation tests to determine the significance of each SNS’s PLV. In other words, we determined if the SNS’s phase preference was significantly higher than the phase preference of the patient’s seizures.

**Figure 5:**
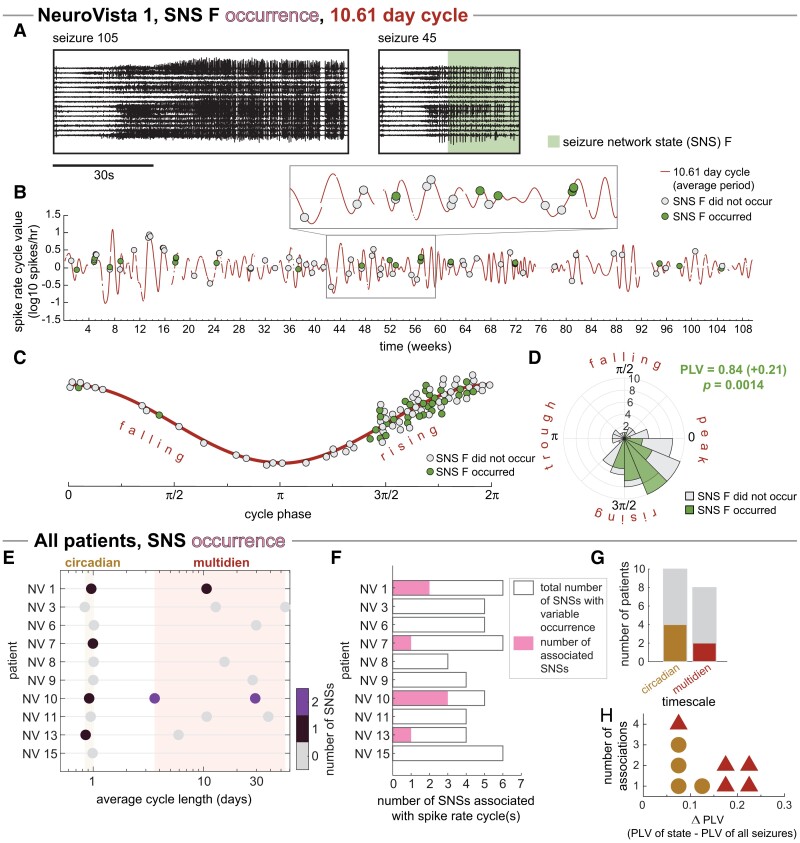
**Associations of seizure network state (SNS) occurrence with spike rate cycles.** (**A–D**) Example patient NeuroVista (NV) 1, SNS F, 10.61-day cycle: (**A**) Example iEEG of seizures with (seizure 105) and without (seizure 45) SNS F (highlighted with green). (**B**) The 10.61-day spike rate cycle is shown in time, with seizures indicated as circles. Circles are coloured by whether the seizure did (green) or did not (grey) contain SNS F. (**C**) The 10.61-day spike rate cycle shown in phase and seizures indicates as before. (**D**) Polar histogram of seizure phases with (green) and without (grey) SNS F. The statistical significance of SNS F’s PLV was determined using a permutation test that randomized which seizures contained SNS F. (**E–H**) All patients: (**E**) Coloured circles indicate the spike rate cycles with significantly associated with one or more SNSs, with statistical significance determined using permutation tests as in (**D**). (**F**) Total number of SNSs associated with spike rate cycles in each patient. Bar outlines provide a reference for the maximum possible number of associated SNSs. (**G**) Summary of patients with circadian or multidien associations. Grey bars indicate the number of patients with each timescale (equivalent to the coloured bars in Fig. 4C).

Figure 5A–D shows an example SNS, SNS F, which preferentially occurred during certain phases of NV 1’s multidien spike rate cycle. In this example, SNS F was most likely to occur during a specific part of the rising phase of the multidien cycle, with the proportion of seizures with this SNS tapering towards the cycle peak. Further, almost all seizures that occurred during the falling phase and the cycle trough lacked this SNS. As such, SNS F’s PLV was significantly stronger than the overall seizure PLV (SNS PLV =0.84, +0.21 relative to PLV of all seizures, p=0.0014) after FDR correction for multiple comparisons.

Across our cohort, four patients (NV 1, 7, 10 and 13) had at least one SNS occurrence that was significantly associated with a spike rate cycle (Fig. 5E and F). The same SNS could be associated with multiple different spike rate cycles (see Supplementary Fig. 4). Four of these patients had an SNS that had a phase preference in their circadian cycle, while two patients had one or more SNS associated with at least one multidien cycle (Fig. 5G). The effect-increase in PLV varied from 0.07 to 0.22 (median: 0.12, Fig. 5H). We interpret these associations as evidence that, in some patients, certain spike rate cycles reveal a modulation in SNS occurrence at a specific timescale, beyond what is explained by seizure occurrence alone.

We then investigated if SNS duration also varied over spike rate cycles. For seizures with a given SNS, we computed the non-parametric circular-linear correlation *D*^45^ between the SNS duration and the phases of the spike rate cycle at which the seizures occurred, using permutation tests to determine statistical significance (see Supplemental Methods). The measure *D* ranges from 0 to 1, with zero indicating no association.

**Figure 6:**
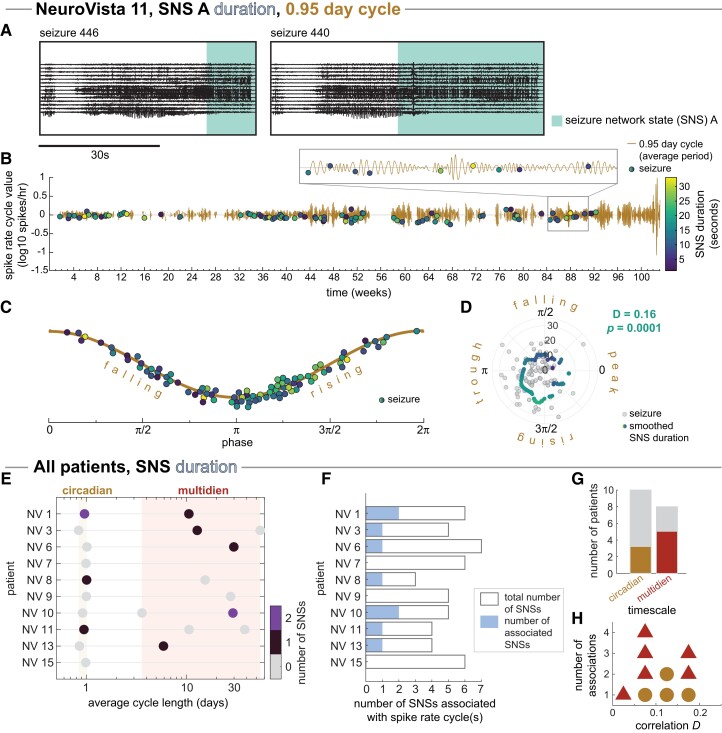
**Associations of seizure network state (SNS) duration with spike rate cycles.** (**A–D**) Example patient NeuroVista (NV) 11, SNS A, 0.95-day spike rate cycle: (**A**) Example iEEG of seizures containing SNS A with varying duration. (**B**) The circadian spike rate cycle is shown in time, with seizures containing SNS A (circles) indicated as circles coloured by SNS duration. (**C**) Representation of the phases of the spike rate cycle. As in (**B**), circles indicate seizures with SNS A, and their colour indicates SNS duration. (**D**) Polar scatter plot of SNS duration versus seizure phase. Each light grey point indicates a seizure, while coloured points correspond to the smoothed seizure duration (π/4 Gaussian window). The significance of the rank linear-circular correlation *D* between SNS A duration and seizure phases was determined using a permutation test that randomly shuffled the seizures’ SNS A duration. (**E–H**) All patients: (**E**) Coloured circles indicate spike rate cycles with one or more significant associations in SNS duration, with significance determined by a permutation test as in (**D**). (**F**) Total number of SNSs that were associated with spike rate cycles in terms of duration in each patient. Bar outlines provide a reference for the maximum possible number of associated SNSs. (**G**) The number of patients that had at least one SNS duration associated with a spike rate cycle at each timescale. Grey bars indicate the number of patients with each timescale. (**H**) Dot plot of the strengths of the significant associations between SNS duration and spike rate cycle phases, measured as non-parametric circular-linear correlation *D*. Marker shape and colour indicate the timescale category of the associated spike rate cycle.

Figure 6A–D shows an example of SNS duration association with a spike rate cycle in NV 11. In this patient, the circadian spike rate cycle was significantly associated with the duration of SNS A after FDR correction for multiple comparisons (p=0.0001). SNS A’s duration was markedly higher during the rising phase than during the falling phase of the spike rate cycle (Fig. 6C); the average duration starts increasing shortly before the trough of the cycle and peaks at approximately 3π/2 in the rising phase before decreasing again (Fig. 6D).

In our cohort, 7 of the 10 patients had one or more SNSs with significantly associated spike rate cycle phases in terms of duration (Fig. 6D and E). Of these patients, three had circadian associations and five had multidien associations (Fig. 6F). The strength of the correlations between SNS duration and spike rate cycle phases varied from 0.04 to 0.18 (median: 0.09) (Fig. 6G). As with SNS occurrence, we interpret these associations as evidence that SNS duration can be modulated over the timescales of spike rate cycles.

To conclude our analysis, we investigated if SNS duration and occurrence are modulated by the same temporal factors (Supplementary Fig. 5). We analysed the coincidence of SNS duration and occurrence modulation by the same factor, such as the same spike rate cycle. Supplementary Fig. 5 shows that this coincidence rate was relatively low and not above chance-level. Finally, we analysed whether certain parts of a seizure (e.g. onset states) were more likely to change over a given timescale. We did not find any significant associations between where the state occurred in the seizure evolution and whether the state was associated with the time since implantation or a spike rate cycle (Supplementary Fig. 6). These results indicate that multiple places in seizure evolutions are susceptible to temporal modulation.

## Discussion

We analysed variability in seizure network states (SNSs) in chronic iEEG recordings, providing novel insight into the patterns and mechanisms of seizure variability. We found that in most patients, SNSs depended on when the seizure occurred in the recording, with some SNSs becoming more or less prevalent and/or increasing or decreasing in duration as the recording progressed. Additionally, several patients had one or more SNSs associated with circadian and/or multidien cycles in interictal spike rate. These associations suggest that seizure features are modulated over multiple timescales, including circadian and multidien timescales that can be revealed by interictal biomarkers.

We first found that seizure evolutions often depended on the amount of time elapsed since the start of the recording. Variability over multiple months to years may reflect non-cyclical changes due to factors such as post-implantation effects,^17,31,32^ medication changes^46^ and slow changes in the epileptic network due to plasticity.^47^ Analysing longer recordings in the future could also determine if persistent seizure variability reflects longer cycles, such as circannual cycles, in brain dynamics.^35,36,48^ Notably, transiently observed SNSs at the beginning of recordings were uncommon in our cohort, suggesting that implantation effects rarely cause atypical seizure network SNSs. Thus, shorter pre-surgical recordings of patients with epilepsy, which typically last for a few days to a few weeks, likely contain a patient’s usual SNSs, although SNS duration and relative SNS prevalence may change over time. Our findings add to the existing literature on variability in brain dynamics across chronic iEEG recordings^17,31,32^ by revealing that multiple features of seizure evolutions also vary across these longer timescales.

Our work builds on past research that provided evidence for seizure variability over specific timescales. For example, it is well-established that in some patients, clinical seizure features such as focal to bilateral tonic-clonic spread are associated with sleep/wake state or day/night cycles.^12-16^ Past analysis of chronic iEEG in canines also discovered shifts in seizure onset patterns as the recording progressed, likely due to post-implantation variability in brain dynamics.^17^ Additionally, variability in seizure onset and spread has been linked to preictal and interictal changes in network features,^22^ band power,^49^ the location of high-frequency oscillations,^3^ and patterns of cortical excitability.^50^ The same interictal features (network dynamics,^51,52^ band power,^53^ high-frequency activity^31^ and cortical excitability^54^) have all been shown to vary over circadian and/or multidien cycles. We now show that seizure evolutions also change over the timescales that influence interictal brain dynamics, suggesting that these fluctuations share common mechanisms. These exact mechanisms are still elusive, and it is also unclear if circadian mechanisms are truly independent or more tied to sleep/wake cycles.^36^

Although we found many associations between seizure timing, spike rate cycles and SNSs, we were unable to explain the full spectrum of SNS variability in our patients. Other approaches could yield more comprehensive and stronger explanations of seizure features. First, our analysis focused on the spike rate phase due to its association with seizure occurrence.^34-36^ However, we also observed that the amplitude of spike rate cycles often varied over time, potentially reflecting variability in the strength of these cycles. Such changes in cycle strength could potentially impact seizure features. Second, as with seizure occurrence,^34,35,37^ different cycles likely interact to produce the observed seizure variability. A predictive model incorporating multiple timescales may be more informative than a single spike rate cycle.^53^ Third, we limited our analysis to each patient’s overall spike rate. Spatial patterns of spike rate also vary over time,^31^ and other interictal events such as high-frequency activity have different temporal profiles than spike rate.^31^ Spatiotemporal variability in interictal dynamics may be linked to seizure variability^3^ and spatial patterns of different interictal events could also be incorporated in multivariate models of seizure features. Other approaches for analysing SNS could also uncover additional modulations; in particular, although a given SNS’s occurrence and duration were often independently modulated, there were likely interactions across SNSs. For example, the duration of an early SNS may depend on whether a seizure progresses to a subsequent possible SNS.^55^ Approaches such as canonical correlation analysis could uncover combinations of seizure features that are associated with combinations of interictal features. Furthermore, for our analysis of SNS occurrence, we asked if the modulation was significantly different to the overall seizure occurrence modulation, thus making it more difficult to detect any weak SNS modulations that followed the same pattern as the seizure occurrence. Finally, we focused on SNSs, as measured by the functional connectivity of the iEEG. Other seizure features may reveal more or additional modulations, which we hope future work will reveal.

Understanding patient-specific seizure variability could provide new clinical strategies for managing seizures in patients with focal epilepsy. First, cycles in seizure features could be added to seizure forecasting algorithms,^56-58^ allowing them to forecast not only when a seizure will occur, but also how the seizure will manifest. Additionally, clinicians could modify a patient’s antiepileptic medication based on both seizure likelihood^57-59^ and seizure severity.^60^ Both interictal and seizure variability may also have implications for treatment efficacy; for example, in a mouse model of temporal lobe epilepsy, optogenetic stimulation only impacted seizures that arose from specific brain states.^10^ Novel, seizure-specific treatments could therefore be designed to fluctuate over the same timescales as the patient’s seizures, thus delivering time-adaptive treatments that account for the patient’s seizure variability. Finally, uncovering the time-varying mechanisms that underlie seizure variability and severity could provide new targets for manipulating seizures and lessening their impact on patients.

While the NV dataset provides an uncommon opportunity to explore seizure variability over longer timescales, it has some limitations. First, due to its small sample size, we were unable to investigate whether there were consistent patterns in seizure modulation across patients. Repeating our analysis in a larger cohort could determine if there are certain characteristic timescales of seizure variability, analogous to chronotypes in seizure occurrence.^15,35,48,61^ Likewise, due to our small sample size and heterogeneous findings, we were unable to compare patient clinical and demographic features, such as age, sex, pathology, or seizure onset zone location, to variability patterns. We also lacked clinical information at the seizure level, such as seizure severity or semiology, which could be related to SNSs and variability patterns. However, other studies have related seizure networks to clinical seizure types.^9,20-22^ Thus, some SNSs likely relate to seizure clinical characteristics, and future studies could use clinical seizure data or quantitative seizure severity markers^62^ to explore this relationship and investigate how seizure symptoms fluctuate over time. Finally, other datasets, such as pre-surgical iEEG recordings, provide better spatial coverage as well as accompanying neuroimaging data. Such data would provide opportunities to relate seizure networks to the patient’s underlying structural connectivity.^63^

In summary, we have shown that features of seizure evolutions vary over multiple timescales within individual patients with focal epilepsy. Like interictal dynamics, seizures can change over the months and years of chronic iEEG recordings as well as over faster timescales, such as circadian and multidien cycles. As with cycles in seizure occurrence, cycles in seizure features can be extracted using the interictal spike rate as a biomarker. Future work could explore whether fluctuations in other interictal features, such as spatial patterns of spikes and high-frequency activity, explain additional seizure features. Ultimately, uncovering the timescales of within-patient seizure variability could lead to new time-adaptive approaches for controlling seizures.

## Supplementary Material

fcad205_Supplementary_Data

## Data Availability

All analysis was performed in MATLAB version R2018b. EMD of interictal spike rate was performed using the MATLAB package CEEMDAN (https://github.com/macolominas/CEEMDAN).^43,44^ The remaining analysis was performed using custom MATLAB scripts. The NeuroVista seizure iEEG data used in this study are available from www.epilepsyecosystem.org. The processed data (NMF *W* and *H* matrices) of all patients is available on Zenodo (DOI 10.5281/zenodo.5503590).
