# Supplementary material for: Chronic intracranial EEG recordings and interictal spike rate reveal multiscale temporal modulations in seizure states
Source: Brain Commun. 2023 Jul 19;5(5):fcad205. doi: 10.1093/braincomms/fcad205 (PMC10484289; doi:10.1093/braincomms/fcad205)
Supplement: fcad205_Supplementary_Data [file fcad205_supplementary_data.docx]

Supplementary Information for:

Chronic intracranial EEG recordings and interictal spike rate reveal multiscale temporal modulations in seizure states

Gabrielle M. Schroeder${}^{1}$, Philippa J. Karoly${}^{2,3}$, Matias Maturana${}^{2,3,4}$,_­­­_
Mariella Panagiotopoulou${}^{1}$, Peter N. Taylor${}^{1,5,6}$,
Mark J. Cook${}^{2}$, Yujiang Wang${}^{1,5,6*}$

1. CNNP Lab (www.cnnp-lab.com), Interdisciplinary Computing and Complex BioSystems Group, School of Computing, Newcastle University, Newcastle upon Tyne, United Kingdom
2. Graeme Clark Institute and St Vincent’s Hospital, University of Melbourne, Melbourne, Victoria, Australia
3. Department of Biomedical Engineering, University of Melbourne, Melbourne, Victoria, Australia
4. Seer Medical Pty Ltd, Melbourne, Victoria, Australia
5. Faculty of Medical Sciences, Newcastle University, Newcastle upon Tyne, United Kingdom
6. UCL Queen Square Institute of Neurology, Queen Square, London, United Kingdom

* Yujiang.Wang@newcastle.ac.uk

# Supplementary Methods

## Seizure iEEG preprocessing

NeuroVista seizure data was previously notch filtered at 50 Hz during the iEEG acquisition and then bandpass filtered (2nd order, zero-phase Butterworth filter from 1-180 Hz) by Karoly et al. (2018). After removing any electrodes with noisy or intermittent signal from the analysis, we re-referenced all iEEG to a common average reference. Time periods with signal dropouts were detected using line length and marked as missing data as in Schroeder et al. (2022).

# Detecting signal dropouts

The NeuroVista data contains time periods of signal dropouts when the iEEG signal was not recorded. We used line length to identify iEEG segments with no signal (i.e., a flat time series with no voltage changes). We defined the line length $L$ of a time series as

$$L=\frac{1}{T-1}\sum_{i=1}^{T-1} \left| x_{i+1}-x_{i} \right|$$

where $x_{i}$ is the $i$th time point in a time series with $T$ time points.

The time-varying line length of each seizure was computed for each iEEG channel in sliding windows (1/10s window, 1/20s overlap). Any time windows with 8 or more channels with line length $\leq$ 0.5, along with the preceding and following time windows, were considered missing data.

## Computing seizure time-varying functional connectivity

Seizure functional connectivity was defined as band-averaged coherence in six frequency bands: delta 1-4 Hz, theta 4-8 Hz, alpha 8-13 Hz, beta 13-30 Hz, gamma 30-80 Hz, high gamma 80-150 Hz. The time-varying coherence in each frequency band was computed for each seizure from onset to termination using a sliding window (10s window, 9s overlap) as in Schroeder et al. (2020). For each 10s window, the band-averaged coherence was calculated using Welch’s method (2s window, 1s overlap, yielding a total of nine subwindows per 10s window). To tolerate some missing data in each seizure, we allowed functional connectivity in each 10s window to be estimated using a subset of the overlapping 2s Welch subwindows. If a 10s time window had five or more 2s subwindows that contained missing data, the seizure was removed from the analysis.

The upper-triangular elements of each symmetric coherence matrix were re-expressed as vectors of length $\left( n^{2}-n \right)/2$, where $n$ is the number of iEEG channels, and each vector was normalised to have an $L1$ norm of 1. Seizure time windows therefore had $6\times\left( n^{2}-n \right)/2$ features describing the pairwise channel interactions in the six different frequency bands.

## Computing progressions of seizure network states (SNS)

To extract seizure network states (SNSs), patterns of recurring functional connectivity were identified in each patient by applying stability NMF (Lee & Seung, 1999; Wu et al., 2016) to all of a patient’s seizure functional connectivity time windows using the same pipeline as in our previous work (Schroeder et al., 2020). This step described each patient’s time-varying seizure functional connectivity using (1) a small number of patient-specific NMF basis vectors that captured patterns of functional connectivity, and (2) time-varying coefficients that denoted the contribution of each basis vector to each time window’s connectivity.

We observed that most seizure time windows had a single NMF basis vector with a high coefficient. As such, a time window’s dominant basis vector (i.e., the basis vector with the highest coefficient) provided a simplified description of the time window’s functional connectivity. Therefore, a seizure’s time-varying functional connectivity could be simplified as a sequence, or progression, of SNSs, where the SNS of each time window was the dominant NMF basis vector. We used this approach to describe each patient’s seizures as progressions of SNSs.

## Preprocessing of interictal spike rate

Detection of interictal epileptiform spikes for this dataset was previously performed and validated (Karoly et al., 2016). The time-varying spike rate for each patient’s recording was summarized as the number of spikes, across all channels, in non-overlapping one hour windows. Due to communication dropouts or failures to regularly store the iEEG data, each one hour segment could have missing segments that affected the spike rate count. To normalize for these dropouts, we normalized each hourly spike rate count by the proportion of captured iEEG data:

$$S_{t,norm}=\frac{S_{t,rec}}{1-D_{t}}$$

where $S_{t,rec}$ is the recorded spike rate (spikes/hr) of hour $t$, $S_{t,norm}$ is the spike rate (spikes/hr) of hour $t$ after normalising for dropouts, and $D_{t}$ is the proportion of dropout time, or missing data, of hour $t$. Recording hours with $D\geq0.75$ were considered missing data.

After normalizing for dropouts, each hourly spike rate $S_{t,norm}$ was log transformed:

$$S_{t}=log10\left( S_{t,norm}+1 \right)$$

yielding the final spike rate, $S_{t}$, of each hour $t$.

Beginning with the shortest missing segments of spike rate data, we then iteratively imputed missing segments of spike rate data using a method similar to Baud et al. (2018). For each missing segment, we first selected the spike rate data segments directly preceding and following the missing segment that were the same length as the missing segment. If this data was available (i.e., did not contain missing values or exceed the endpoints of the recording), we used the surrounding segments to generate spike rate data for the missing segment. This data was generated by linearly interpolating between the means of the surrounding segments and then adding Gaussian noise with a mean of zero and standard deviation of the surrounding segments. Any resulting interpolated data with a spike rate of less than zero was changed to zero. The length of each interpolated segment was also recorded so that interpolated data was only included in the analysis when its length was much shorter ($\leq20\%$) than the period of the analysed spike rate cycle. Any remaining missing time points were temporarily set to the mean spike rate value prior to EMD and then returned to missing values after the decomposition and Hilbert transform.

## Extracting interictal spike rate cycles using empirical mode decomposition (EMD)

Empirical mode decomposition (EMD) (Huang et al., 1998) was then used to extract intrinsic mode functions (referred to as “spike rate cycles" in the Results) from each spike rate time series. Briefly, EMD decomposes any given signal into a set of signals known as intrinsic mode functions (IMFs) that exactly reconstruct the original signal when summed together along with a with a residual signal. A key property of each IMF is that it must have approximately the same number (up to +/- 1) of extrema as zero-crossings, which ensures that there are no riding waves in the extracted IMFs, as well as a local mean (i.e., the mean of the maximal and minimal envelopes of the IMF) of zero. This property also ensures a well-defined Hilbert transform, allowing us to extract the phase of the signal fluctuations.

We used a variation of EMD known as CEEMDAN (Torres et al., 2011; Colominas et al., 2014) that helps ensure that each IMF contains oscillations with a similar timescale (i.e., the mode’s period does not dramatically vary over time) by adding noise to the time series prior to the decomposition. The standard deviation of the added noise was scanned from 0.0025 to 0.125 in steps of 0.0025, and the decomposition at each noise level was performed with 100 noise realizations, a maximum of 1000 sifting iterations to extract each mode, and the signal-to-noise ratio increasing for every stage of the decomposition. This initial step yielded 50 versions, one for each noise level, of the EMD decomposition for each patient’s spike rate time series.

For each decomposition, we used the Hilbert transform to determine the time-varying frequency, phase, and amplitude of each extracted spike rate IMF (Huang et al., 1998). We initially estimated the average period of each IMF using the median frequency of only the original (i.e., non-interpolated) spike rate data, excluding the first and last ten days of the recording due to possible instability in the frequency estimate at the time series boundaries. For each IMF, segments with interpolated spike rate were removed if their duration exceeded 20% of the IMF’s period. To define each IMF’s timescale, we then recomputed the average period of each IMF as above, now using all of the IMF’s non-missing data. The average amplitude of each IMF was computed using the same process.

For each noise level, we then computed the pairwise index of orthogonality, $O$ (Huang et al., 1998), between all pairs of time series from the EMD decomposition (i.e., the IMFs and the residue signal):

$$O_{i,j}=\frac{1}{T}\sum_{t=1}^{T} \frac{C_{i}\left( t \right)C_{j}\left( t \right)}{C_{i}\left( t \right)^{2}+C_{j}\left( t \right)^{2}}$$

where $C_{i}\left( t \right)$ is the $i$th extracted time series, $C_{j}\left( t \right)$ is the $j$th extracted time series, $t$ is the time point in each time series, and $T$ is the total number of time points with spike rate data in both time series. The normalization by $\frac{1}{T}$ allowed us to compare $O$ across pairs of time series that had different amounts of missing data due to the spike rate interpolation step. $O$ is close to zero when the two time series are locally orthogonal (i.e., do not contain oscillations at similar frequencies during the same time interval). Thus, to minimize overlap in the frequencies of different spike rate cycles, we found the maximum absolute value of the pairwise $O$ for each decomposition and then selected the noise level that minimized this value. This decomposition was used for all downstream analysis.

The median amplitudes versus median periods of the IMFs of the selected decompositions are shown in Supplementary Fig. 3. To focus our analysis on the primary, robust contributors to spike rate cycles, we limited our analysis to IMFs with locally prominent amplitudes that had median periods that were less than a quarter of the duration of the patient’s recording (see Supplementary Fig. 3). Across patients, we observed a clear distinction between cycles with median periods of approximately one day (0.83 to 1.03 days) and cycles with longer periods (3.93 to 54.77 days). We labeled these timescales as circadian and multidien cycles, respectively.

## Supplemental comparisons

In addition to the main text statistical analysis, we also compared (1) the overall spike rate, prior to EMD, to SNS occurrence and SNS duration using Wilcoxon rank sum tests and Spearman’s correlation, respectively, and (2) overall seizure duration to time since implantation and spike rate cycles using Spearman’s correlation and rank circular-linear correlation, respectively. These tests were included in the overall FDR correction for multiple comparisons (see main text Methods, "Statistical analysis") and the results are presented in Supplementary Fig. 4.

# Supplementary Table 1: Patient metadata

Supplementary Table 1 provides the following metadata for the NeuroVista patients:

- **Age (yrs):** patient age in years.
- **Sex:** patient sex.
- **Age at diagnosis (yrs):** patient age when they were diagnosed with epilepsy, in years.
- **Lobe:** purported lobe of onset of the patient’s seizures, based on clinical findings. Note that some patients had seizures arising from multiple lobes (e.g., OP = occipital/parietal onset).
- **Previous resection**: whether the patient had undergone surgical resection prior to the chronic recording.
- **# seizures analyzed:** number of the patient’s seizures analyzed in this work.
- **# electrodes analyzed:** number of recording electrodes included in the analysis after removing noisy electrodes.
- **Total recording time (days):** total duration of the intracranial recording time, in days.
- **Sampling frequency:** sampling frequency at which intracranial data was acquired and stored.


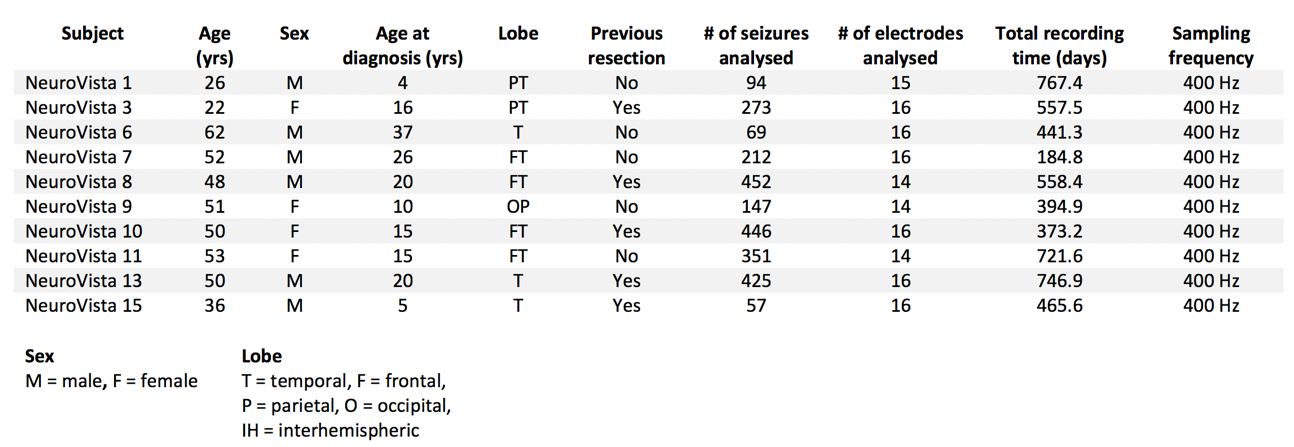


**Supplementary Table 1**

# Supplementary Figure 1: Seizure network states (SNSs) of an example patient, NeuroVista 1

Supplementary Fig. 1A shows the six network SNSs of NeuroVista 1. Each SNS was derived using stability NMF (Wu et al., 2016), as in Schroeder *et al.* (2020). Additionally, Supplementary Fig. 1B provides a summary of each SNS, as in Fig. 1B in the main text. For each SNS, the summary visualization was created by computing the eigenvector centrality of each functional connectivity matrix; for example, the first column of each heatmap in Supplementary Fig. 1B is the eigenvector centrality of the delta functional connectivity matrix of the corresponding SNS. Eigenvector centrality has previously been used to characterize seizure network interactions (Burns et al., 2014) and, in this application, represents the primary group of channels with high coherence in each frequency band. For example, in SNS A, we see that the strongest connections are between a pair of channels in gamma and high gamma (Supplementary Fig. 1A, first row). The same pair of channels has high eigenvector centrality (Supplementary Fig. 1B., top heatmap).

We also note that SNSs are not orthogonal and can therefore share some common elements; for example, the strong connections in SNS B in the gamma band are also present in SNS C in the gamma band. However, SNS C has additional strong gamma connections, as well as different connectivity patterns in other frequency bands, that distinguish it from SNS B. Across patients, the median and maximum Pearson correlation between the connectivity of pairs of SNSs ranged from 0.05 to 0.47 and 0.32 to 0.80, respectively.


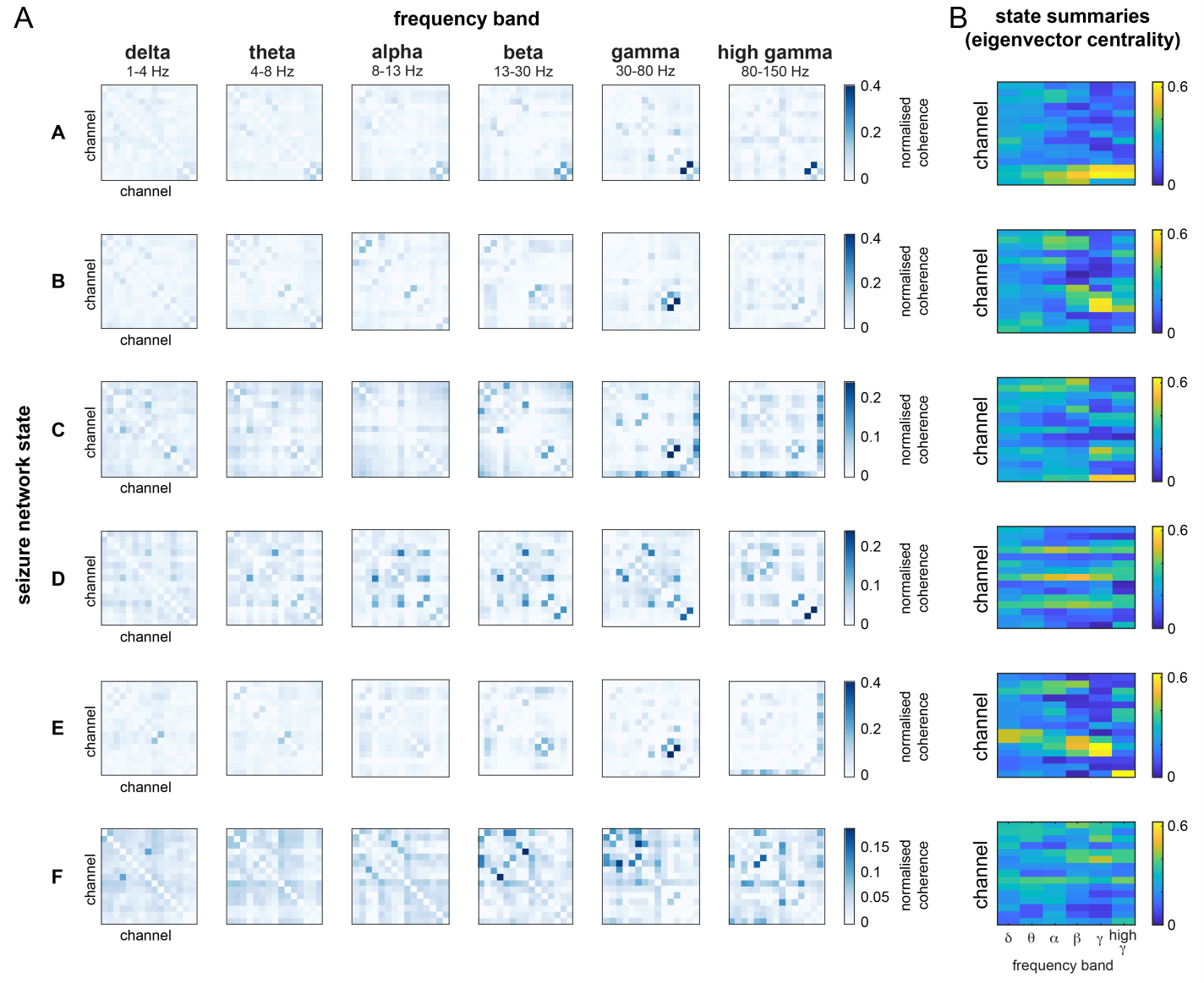


**Supplementary Fig. 1: Seizure network states (SNSs) of NeuroVista 1.** Rows correspond to SNSs. A) Each SNS describes the coherence between pairs of iEEG channels in six frequency bands (columns). Thus, each SNS is composed of six functional connectivity matrices, one for each frequency band. Colormap limits are consistent within each SNS (i.e., across each row). Self-connections (diagonal matrix elements) are not shown. B) Summaries of each SNS, created by computing the eigenvector centrality of each connectivity matrix in (A). Each column corresponds to the eigenvector centrality of one functional connectivity matrix.

# Supplementary Figure 2: First and last occurrences of seizure network states (SNSs) in patient recordings

Supplementary Fig. 2 shows the first and last known occurrences of each SNS in each patient. Each SNS visualization is also colored by whether the SNS’s occurrence was significantly associated with the time since the start of the recording. As a reminder, SNSs are patient-specific, even if they share the same letter label. In most cases, the time from first to last occurrence of a SNS spans the majority of the patient’s recording. Thus, most SNSs are not limited to a specific section of the recording.

Note that due to missing and noisy data, some of the patient’s seizures were not captured in the iEEG recordings. As such, some SNSs may have occurred earlier or later in the recording period than displayed here. As such, this analysis can only conclusively say when SNSs *did* occur, and there is some uncertainty regarding when SNSs *did not* occur.


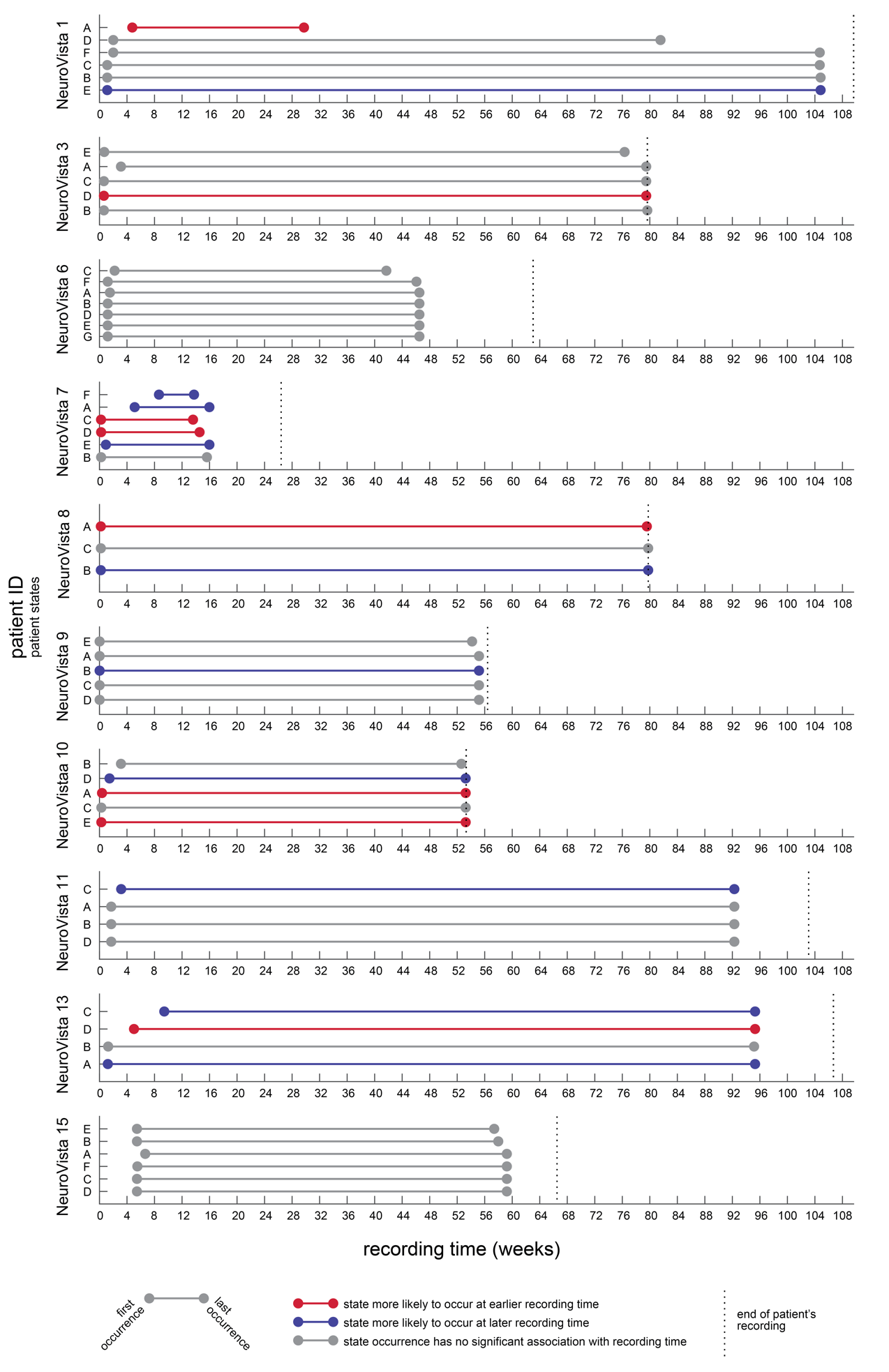


**Supplementary Fig. 2: First and last known occurrences of each seizure network state (SNS) in each patient.** In each patient, the times in the recording of the first and last occurrences of each SNS (circles), with the time spanned by those occurrence marked with horizontal line. SNSs are ordered from the shortest to longest amount of time that they spanned. SNS markers are colored by whether the SNS’s occurrence was significantly associated with the time since implantation, with significance determined by Wilcoxon rank sum tests . Significant p-values are defined as p < 0.05 after FDR correction for multiple comparisons.

# Supplementary Figure 3: EMD of interictal spike rate and selection of spike rate cycles for comparison with seizure features

For each patient, we used Empirical Mode Decomposition (EMD) (Huang et al., 1998; Torres et al., 2011; Colominas et al., 2014) to extract cycles in interictal spike rate. Supplementary Fig. 3 shows the median amplitudes versus median periods (days/cycle) of each EMD cycle, or IMF (see Supplementary Methods). We limited our analysis to cycles that had both (1) locally prominent amplitudes, which we defined as local maxima (found using MATLAB function *findpeaks*) in these plots with amplitudes greater than or equal to 20% of the patient’s highest median IMF amplitude and (2) median periods of less than a quarter of the recording’s duration. These cycles are shown using the colored circles in each patient. The fastest IMF of each patient was not included in the analysis since these oscillations are often thought to contain temporally unstructured noise.


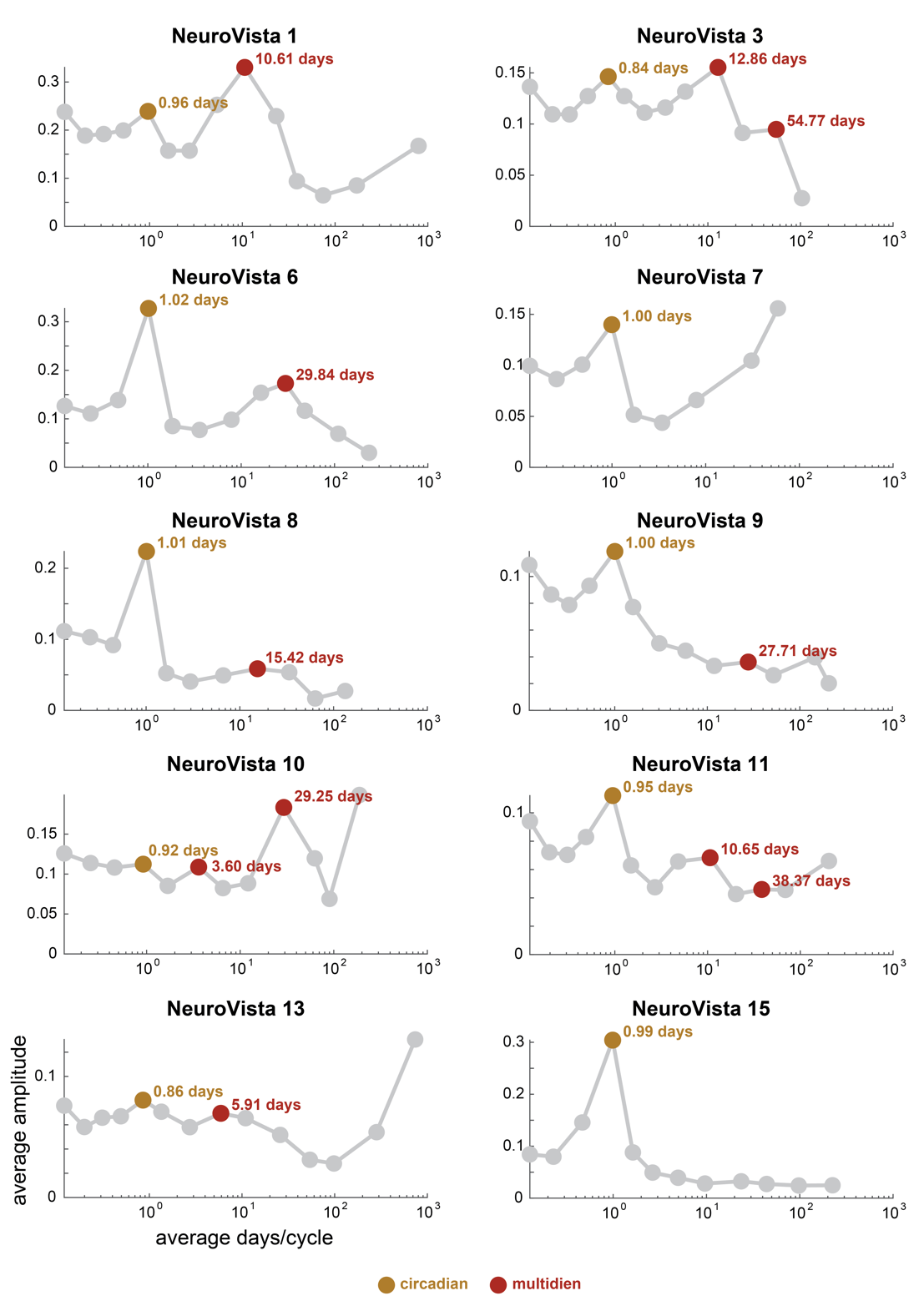


**Supplementary Fig. 3: Amplitudes and periods of the spike rate cycles extracted using EMD.** Each plot shows the median amplitude and period of all of the extracted spike rate cycles (circles) of each patient. Colored circles indicate cycles that were analyzed in this study, with the color indicating the timescale category of the cycle (circadian or multidien). Spike rate residues are not shown.

# Supplementary Figure 4: Supplementary spike rate, seizure duration, and seizure network state (SNS) analyses and visualizations

Supplementary Fig. 4 shows which SNSs and whether total seizure duration were significantly associated with seizure time since implantation, spike rate cycles, and spike rate in each patient. We emphasize that SNSs are *not* comparable across patients, even if they share the same label and color.

The first columns in Supplementary Fig. 4A and4B show which SNSs were significantly associated with overall spike rate after FDR correction for multiple comparisons. While overall spike rate was often associated with these seizure features, it was usually associated with the same or fewer SNSs than specific timescales (spike rate cycles and time since implantation) (Supplementary Fig.4). The only exceptions were the occurrence of SNS C in NeuroVista 1, the durations of SNSs C and D in NeuroVista 6 and, the duration of SNS C in NeuroVista 10, which were only associated with overall spike rate. Cases where overall spike rate, but not individual spike rate fluctuations, are associated with SNSs may be due to spike rate features that our analysis did not capture (e.g., cycle amplitude) or joint effects of modulations over different timescales that were not significant at the level of individual timescales.

Supplementary Fig. 4B also indicates when a patient’s total seizure duration was associated with overall spike rate, spike rate cycles, and/or recording time. Seizure duration was only associated with spike rate cycles in two patients and recording time in four patients; thus, SNS duration associations were more widespread than seizure duration associations in our cohort.


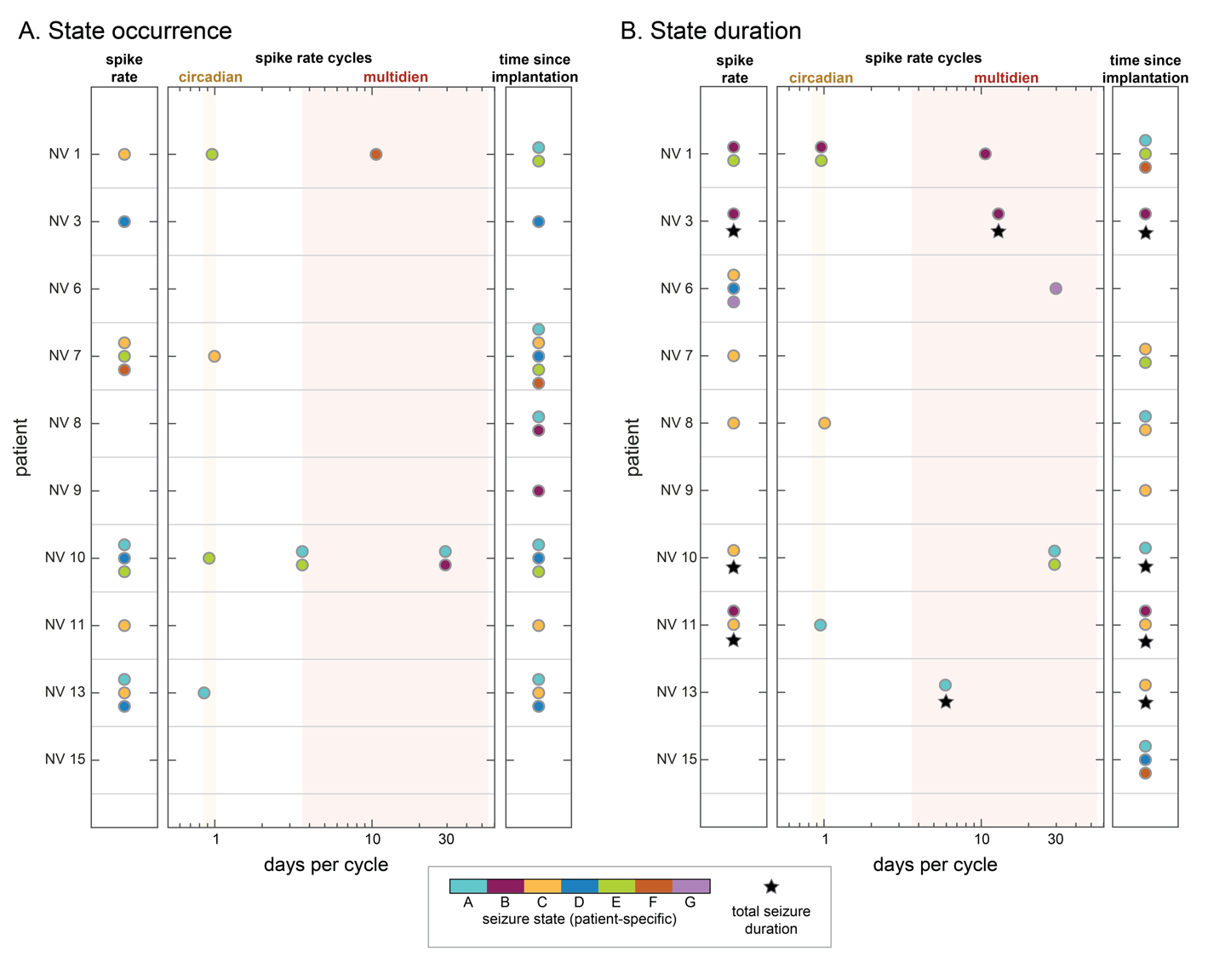


**Supplementary Fig. 4: Seizure network states (SNSs) significantly associated with recording time, spike rate cycles, and spike rate.** Colored circles indicate the SNSs with occurrences (A) or duration (B) significantly associated with overall spike rate (left columns), spike rate cycles (middle columns), and recording time (right columns). Rows for different patients are demarcated by horizontal grey lines. SNSs are not comparable across patients, but are comparable within each patient. In (B), associations with total seizure duration are also marked with a black star. For SNS occurrence, significant associations were determined using Wilcoxon rank sum tests for overall spike rate and recording time; and permutation tests to generate null phase locking value (PLV) distributions for spike rate cycles. For SNS duration and total seizure duration, significant associations were determined using Spearman correlation and the corresponding test statistic for overall spike rate and recording time; and permutation tests to generate null rank linear-circular correlation distributions for spike rate cycles. Significant p-values are defined as p < 0.05 after FDR correction for multiple comparisons. See main text Methods (“Statistical analysis”) and Supplementary Methods (“Supplemental comparisons”) for more details.

## Supplementary Figure 5: A seizure network state’s occurrence and duration are usually independently modulated

In previous work, we found that seizure duration could vary independently of changes to the seizure’s network evolution (Schroeder et al., 2022). This finding suggests that features that characterize seizure network evolutions (e.g., SNS occurrence) and features that influence seizure duration (e.g., SNS duration) could have different modulators. We therefore next asked whether a given SNS’s occurrence and duration were associated with the same timescale, which we would interpret as co-modulation by the same time-varying process.

In each patient, we first determined whether each SNS’s 1) occurrence, but not duration, was associated with the time since implantation, 2) duration, but not occurrence, was associated with the time since implantation, or 3) occurrence and duration were both associated with time since implantation. Supplementary Fig. 5A shows the number of SNSs in each patient that belonged to each category. Although the occurrence and duration of the same SNS were sometimes both associated with time since implantation, it was more common for only one to be associated with time since implantation.

We repeated this analysis for SNS associations with spike rate cycles (Supplementary Fig. 5B). Note that for this analysis, a SNS could belong to multiple categories if its occurrence and duration were associated with different spike rate cycles; for example, a SNS’s occurrence could be associated with a circadian cycle (category 1) while its duration could vary over a multidien cycle (category 2). For spike rate timescales, a SNS’s occurrence and duration were only associated with the same timescale in one SNS in NeuroVista 1 and one SNS in NeuroVista 10 (Supplementary Fig. 4). Thus, joint modulation of SNS occurrence and duration was rare over circadian and multidien timescales.


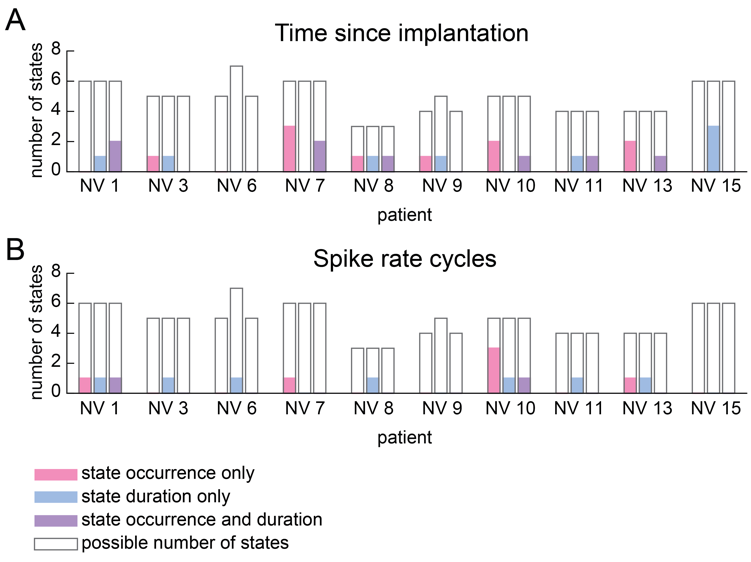


**Supplementary Fig. 5:** **Independent and coinciding timescales of SNS occurrence and SNS duration modulation** A) From left to right, the three bars for each patient show the number of the patient’s SNSs in which 1) only SNS occurrence was associated with time since implantation (pink), 2) only SNS duration was associated with time since implantation (blue), and 3) occurrence and duration were both associated with time since implantation (purple). Grey outlines provide a reference for the maximum number of SNSs that can belong to each category (i.e., for occurrence counts, the number of SNSs with variable occurrence, and for duration counts, the total number of SNSs) in each patient. B) Same measures as (A) for SNS associations with spike rate cycles.

# Supplementary Figure 6: Locations of modulated SNSs in SNS evolutions

We also investigated whether modulated SNSs tended to occur during certain parts of seizure evolutions. In each patient, we first identified the order in which SNSs occurred in each seizure. Only the first occurrence of a SNS was considered; for example, in the hypothetical SNS progression

$$BBBAAACCACCCEEEEEE$$

SNS $B$ would be first, $A$ would be second, $C$ would be third, and $E$ would be fourth. This seizure does not provide any information about other SNSs, such as SNS $D$. A SNS’s *location* was then defined as the SNS’s most common (i.e., mode) order of occurrence across all the patient’s seizures that included the SNS. Supplementary Fig. 6A shows the distributions of SNS locations in each patient. Across all patients, we then computed the mean SNS location for four categories of SNSs (Supplementary Fig. 6B, top row, left to right): (1) SNSs whose occurrence was associated with seizure recording time, (2) SNSs whose duration was associated with seizure recording time, (3) SNSs whose occurrence was associated with at least one spike rate cycle, and (4) SNSs whose duration was associated with at least one spike rate cycle.

We then performed permutation tests to determine whether significant SNSs occurred earlier or later in the seizure evolutions than expected by chance (Supplementary Fig. 6B, bottom row). For each scenario, we permuted SNS labels in each patient, selected the same number of “significant" SNSs and their corresponding locations from each patient, and then recomputed the mean SNS location across all patients. Thus, this permutation test accounts for the distribution of SNS locations in each patient. For each test, the null distribution of mean SNS locations was computed using 10,000 permutations. The $p$-value of the observed mean location was defined as the percentage of permutations with a mean location as extreme (i.e., equidistant from the center of the distribution) as the observed mean location. In all scenarios, the mean SNS location did not significantly differ from chance, suggesting that SNSs that occurred earlier or later in seizure evolutions were not preferentially modulated.


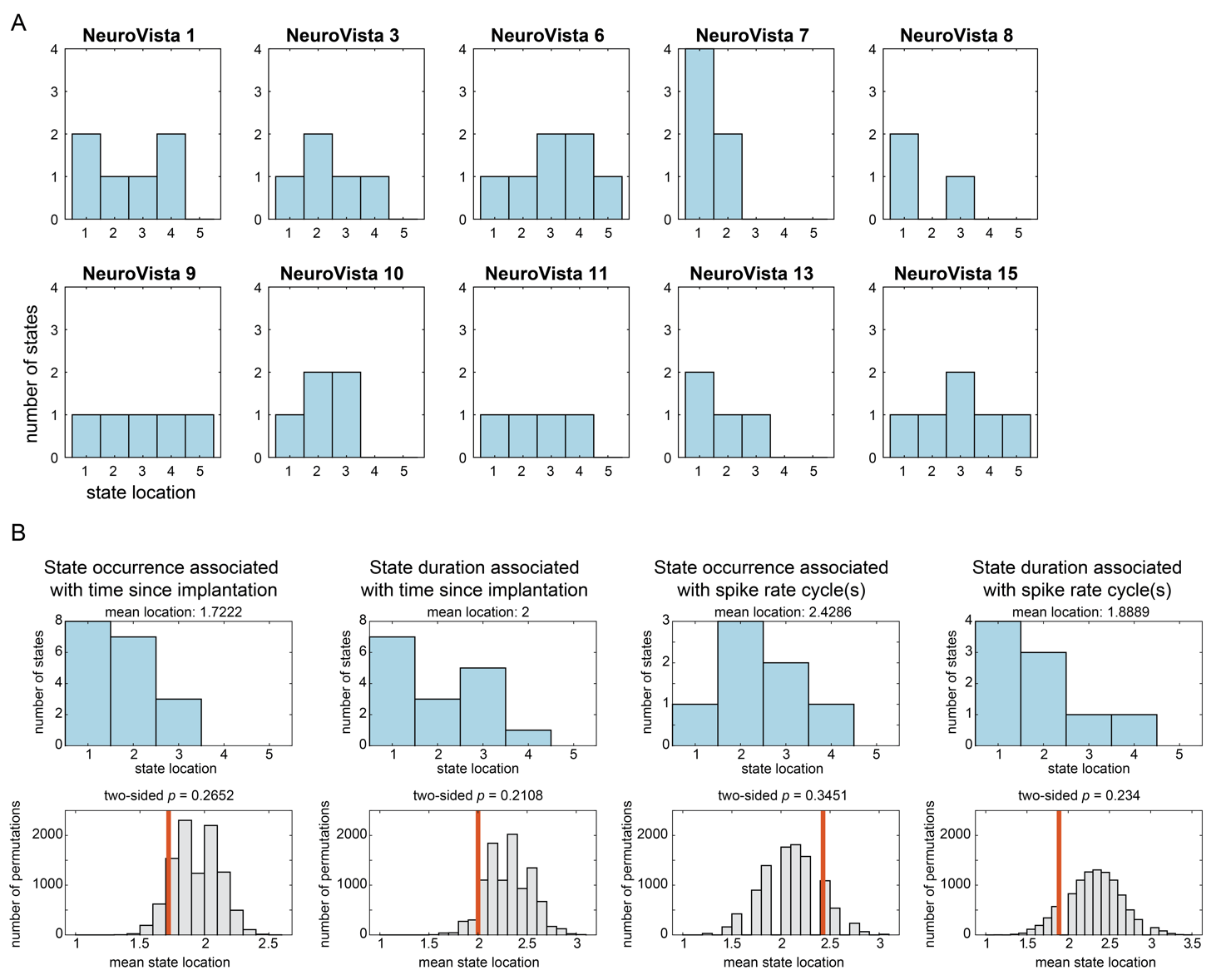


**Supplementary Fig. 6: Locations of significant seizure network states (SNSs) in SNS evolutions.** A) Distributions of SNS locations in each patient. B) SNS locations of SNSs significantly associated with seizure recording time and spike rate cycles, with significance determined by permuting SNS labels in each patient. Top row: distributions of locations of significantly associated SNSs. Bottom row: permutation test results for determining the significance of the mean SNS location in each scenario. The observed mean SNS location is marked with a red line in each distribution of permuted mean SNS locations.

**Supplementary References**

Baud MO, Kleen JK, Mirro EA, et al. Multi-day rhythms modulate seizure risk in epilepsy. *Nature Communications*. 2018;9(88):1-10. doi:[10.1038/s41467-017-02577-y](https://doi.org/10.1038/s41467-017-02577-y)

Burns SP, Santaniello S, Yaffe RB, et al. Network dynamics of the brain and influence of the epileptic seizure onset zone. *Proceedings of the National Academy of Sciences*. 2014;111(49):E5321-E5330. doi:[10.1073/pnas.1401752111](https://doi.org/10.1073/pnas.1401752111)

Colominas MA, Schlotthauer G, Torres ME. Improved complete ensemble EMD: A suitable tool for biomedical signal processing. *Biomedical Signal Processing and Control*. 2014;14:19-29. doi:[10.1016/j.bspc.2014.06.009](https://doi.org/10.1016/j.bspc.2014.06.009)

Huang NE, Shen Z, Long SR, et al. The empirical mode decomposition and the Hubert spectrum for nonlinear and non-stationary time series analysis. *Proceedings of the Royal Society A: Mathematical, Physical and Engineering Sciences*. 1998;454:903-995. doi:[10.1098/rspa.1998.0193](https://doi.org/10.1098/rspa.1998.0193)

Karoly PJ, Freestone DR, Boston R, et al. Interictal spikes and epileptic seizures: Their relationship and underlying rhythmicity. *Brain*. 2016;139:1066-1078. doi:[10.1093/brain/aww019](https://doi.org/10.1093/brain/aww019)

Karoly PJ, Kuhlmann L, Soudry D, Grayden DB, Cook MJ, Freestone DR. Seizure pathways: a model-based investigation. *PLoS Computational Biology*. 2018;14(10):e1006403. doi:[10.26188/5b6a999fa2316](https://doi.org/10.26188/5b6a999fa2316)

Lee DD, Seung HS. Learning the parts of objects by non-negative matrix factorization. *Nature*. 1999;401:788-791. doi:[10.1038/44565](https://doi.org/10.1038/44565)

Schroeder GM, Diehl B, Chowdhury FA, et al. Seizure pathways change on circadian and slower timescales in individual patients with focal epilepsy. *Proceedings of the National Academy of Sciences*. 2020;117(20):11048-11058. doi:[10.1073/pnas.1922084117](https://doi.org/10.1073/pnas.1922084117)

Schroeder GM, Chowdhury FA, Cook MJ, et al. Multiple mechanisms shape the relationship between pathway and duration of focal seizures. *Brain Communications*. 2022;4(4). doi:[10.1093/braincomms/fcac173](https://doi.org/10.1093/braincomms/fcac173)

Torres ME, Colominas MA, Schlotthauer G, Flandrin P. A complete ensemble empirical mode decomposition with adaptive noise. In: *2011 IEEE International Conference on Acoustics, Speech and Signal Processing (ICASSP)*.; 2011:4144-4147. doi:[10.1109/ICASSP.2011.5947265](https://doi.org/10.1109/ICASSP.2011.5947265)

Wu S, Joseph A, Hammonds AS, Celniker SE, Yu B, Frise E. Stability-driven nonnegative matrix factorization to interpret spatial gene expression and build local gene networks. *Proceedings of the National Academy of Sciences*. 2016;113(16):4290-4295. doi:[10.1073/pnas.1521171113](https://doi.org/10.1073/pnas.1521171113)
